# Observations on Epilepsy
*Read at a meeting of the Bristol Medico-Chirurgical Society, on 14th May, 1930.


**Published:** 1930

**Authors:** J. V. Blachford

**Affiliations:** Physician in charge of the Department of Mental Diseases, Bristol General Hospital; late Medical Superintendent, Bristol Mental Hospital


### The Bristol Medico-Chirurgical Journal

"*Scire est nescire, nisi id me
Scire alius sciret.*"

AUTUMN, 1930.

### OBSERVATIONS ON EPILEPSY.*

BY

J. V. BLACHFORD, C.B.E., M.D.,

*Physician in charge of the Department of Mental Diseases, Bristol General Hospital; late Medical Superintendent, Bristol Mental Hospital.*

THE term epilepsy, from ἐπίληψις a seizure, or taking hold of, formerly had a very definite meaning, and was applied only to those cases in which there was a sudden attack of unconsciousness accompanied by convulsions. Of late years, however, it has been used in connection with so many attacks, including migraine, narcolepsy, pyknolepsy, vaso-vagal and even occasional slight syncopal attacks, that the best definition would appear to be a temporary paroxysmal disorder, not hysterical, of any part of the nervous system. Thus there may be as many epilepsies as there are parts of that system, the symptoms depending upon the part implicated, and as it has been suggested

* Read at a meeting of the Bristol Medico-Chirurgical Society, on 14th May, 1930.

N

that even the sympathetic ganglia may be subject to epilepsy, the definition cannot be confined to the central nervous system. The object of this paper, however, is to discuss that form, the old "falling sickness" or so-called idiopathic epilepsy, which is characterized by loss of consciousness and convulsive seizures, and frequently associated with various forms of mental derangement.

The three points that I propose to consider are: (1) The possible or probable cause of the attack; (2) the seat of origin of the impulses which originate the convulsion; (3) the relation of the seizures to the mental disturbances occurring immediately before, after and between the attacks.

James Collier, in his Lumleian Lectures in 1928, discusses the probable cause of this disease, and suggests that the irritant causing the attack is probably a toxin formed in some metabolic dyscrasia, that this particular toxin may not be confined to those suffering from epilepsy, but that in them some part of the central nervous system is congenitally weak and susceptible to the stimulant effects of the poison. In support of this theory he quotes the results of experiments on animals, by which it was shown that when certain poisons were administered a smaller dose was required to produce convulsions in those in which the central nervous system had been damaged than in animals in which it was intact. He says also that an endocrine origin has been suggested, but thinks the theory he has advanced the more probable.

W. G. Lennox and Stanley Cobb have dealt very exhaustively with the question of the origin of the seizures, and after analysing the statistics of various writers and the results of experiments, conclude that at present nothing is known as to the exciting cause.

In certain cases of epileptiform seizures they think that acidosis acts as a sedative to nerve cells, alkalosis renders them more excitable. They find that abstinence from meat has no beneficial effect, but in some cases fasting lessens the number of seizures. They appear to suggest that an ordinary amount of meat with plenty of fat and cream is more likely to be beneficial than a carbohydrate diet.

I have come across one case, a male epileptic, whose fits are always worse after taking eggs. On one occasion, while taking luminal, he had been going on very well for some time, but about four hours after having an egg he had a strong fit, and he told me that he had not had an egg before for more than twelve months, as they always made him worse.

Though numerous experiments have been tried and observations made, the subject seems far from being settled, but their periodicity, the fact that as a rule they commence at that period of life when certain endocrine glands are becoming mature, that once started they tend to continue throughout life, and that the effects of dieting are so disappointing, all point to an endocrine origin, the trouble being due, perhaps, to the excessive secretion of a hormone; or (what is more probable) the secretion is normal, but the part of the nervous system implicated is, as Collier suggests, congenitally weak and susceptible.

The next question is, which part of the central nervous system is responsible for starting the convulsions? Hughlings Jackson, in his Lumleian Lectures in 1890, said he was of opinion that the primary discharge was from the cells of the highest level, probably those in front of the Rolandic area, which he looked upon as the highest motor cells, in other words the cells which originate the most complex groups of movements. Other neurologists have suggested various sites, from the cord to the cortex; but the majority still hold that the discharge occurs in some part of the cortex, the clonic character of some of the convulsions being adduced as evidence. That the cortex is involved can scarcely be in doubt, but there appears to be a good deal of evidence in favour of its implication being secondary to impulses arising in some other part of the nervous system, probably the corpus striatum.

In the first place, those seizures which are known to originate in the cortex, constituting cortical or Jacksonian epilepsy, differ materially from the major attacks, which are characteristic of so-called idiopathic epilepsy. The twitching of the muscles spreads deliberately, seldom involving those of the whole body, consciousness is rarely lost completely, and when it is lost this occurs late in the attack, whereas in the idiopathic form consciousness is lost before the convulsions commence, and these become general so quickly that it is almost impossible to follow the sequence of events.

In an affection in which symptoms of mental alienation, due to cortical derangement, are so common, were the cortex the primary seat of the trouble we should expect those symptoms to occur even more frequently than they do, and to arise earlier in the course of the case.

In general paralysis seizures indistinguishable from those of epilepsy frequently occur. At one time it was thought that the characteristic cry was the distinguishing feature of epilepsy; yet I have heard it at the commencement of an epileptiform convulsion in a general paralytic, and it is often absent from a true epileptic attack. Now, although this disease affects every part of the nervous system, in the majority of cases, if not in all, it commences in the cortex, and is fairly advanced there before showing evidence of the involvement of deeper structures. If the seizures are cortical in origin they should occur early, but as a rule they do not, and when they do the prognosis as regards life is grave, the case running a rapidly fatal course, showing that the centres more deeply seated were attacked early.

The epileptiform attacks in cases of alcoholism do not occur early, as we should expect did they arise primarily in the cortex, alcohol affecting that region first. As a rule, they come on quite late in the case, and though some patients addicted to alcohol become confirmed epileptics, this only occurs after a long period of intemperance.

A certain proportion of cases of epilepsy commence late in life, when there is every reason to think it may be due to arterial degeneration. In these again the seat of the mischief does not appear to be the cortical cells, for many senile dements show marked changes in this region who have never had a seizure. I can call to mind one in whom almost the whole frontal cortex had atrophied and the patient talked incoherent nonsense, but had never had a convulsion. The evidence in these cases seems to point to general epileptiform convulsions having their seat of origin in some other part than the cerebral cortex.

Some years ago I searched the post-mortem records of the Bristol Asylum for the previous twenty years to discover all cases in which a lesion strictly limited to the basal ganglia had been found, and compared the results with the notes taken during life. There were seventeen in all, and were as follows :—

| *Site of Lesion.* | | *Clinical Picture.* | |
|---|---|---|---|
| Optic thalamus .. | 3 | Dementia, no paralysis, no convulsions .. .. | 3 |
| Optic thalamus and corpus striatum.. | 3 | Dementia .. .. .. | 1 |
| | | Seizures, paralysis, dementia, speech much affected .. .. .. | 1 |
| | | Seizure with strong convulsions.. .. .. | 1 |
| Corpus striatum .. | 10 | Speech affected .. .. | 4 |
| | | Hallucinations of sight and hearing .. .. | 1 |
| | | Epilepsy, dementia, destructiveness .. .. | 1 |
| | | Fits two or three years before admission .. | 1 |
| | | Left-sided convulsions.. | 1 |
| | | Epileptiform fits .. .. | 1 |
| | | Pupils normal, knee-jerks present .. .. .. | 1 |

In one of the cases in which speech alone was affected the patient, a woman nearly seventy years of age, had several transient attacks of aphasia, in which she was slightly giddy, and could not remember words in the middle of conversation. Post-mortem, the only lesions found were several small spots of softening in the corpus striatum. From this table it seems that lesions of the thalamus alone do not give rise to any motor trouble, but that lesions of the corpus striatum are frequently accompanied by convulsions or aphasic attacks, or both.

Acute distension of the lateral ventricles with fluid will also cause general convulsions. The following case may be of interest in this connection.

A female, aged 43, was admitted to the Bristol Asylum on 13th October, 1919. She was single, and suffering from mania and epilepsy. The following history was obtained from her brother and sister :—

When two years old she had convulsions, but soon recovered and was apparently normal till she was seventeen, when she had a bad breakdown, apparently an attack of manic-depressive insanity, and was in bed for eight weeks or more at home. Her chief delusion was that they were going to bury her alive. She recovered from this, but had a similar attack when she was twenty-two, and another when twenty-six; between them she was facile and childish, but of a very nice disposition, always willing to help anyone. Sometime in the year 1916, when about forty, she became strange, at times complaining of pain in her head, and on several occasions when sitting or talking to her brother or sister it was noticed that she became pale, extended her legs, and turned her head and eyes up and to one side. She did not appear to lose consciousness completely, however, though for a few seconds after the turns she was lost and confused. She became so dirty in her habits, quarrelsome and disagreeable to those around her, that her relatives were compelled to have her placed under restraint. On admission she was very irresponsible and with little memory for recent events, thought her friends were trying to defraud her, and was disagreeable and rude if addressed.

On 19th October she had a series of epileptiform seizures and was unconscious. By the 21st she had had about two hundred attacks, but had regained consciousness and complained of pain in her head. On 25th October she was much better, but had pain in the calf of the left leg and the left tendo achillis seemed unduly rigid. On 9th November she complained at times of pain in the head and noises in the right ear. On 10th June, 1920, she is noted as giving no trouble; on 21st October, 1921, as maintaining her physical condition, and very cheerful; on 25th March, 1922, as being childish and good-natured and doing some sewing in the ward. After this she went on very well, did useful sewing, was happy and agreeable to those around her, and was about to be sent to her friends on trial, when she developed an attack of pneumonia from which she died on 22nd December, 1922.

At the post-mortem examination the pia-arachnoid was found thickened but stripped easily. The posterior part of the callosal convolution on the right side, as also the right hippocampal gyrus, was yellow and soft, and the posterior part of the corpus callosum was wasted and yellow. The ventricular ependyma was granular, and in the right choroid plexus was a fair-sized, shrivelled mass which, by its pressure, had caused considerable atrophy of the right corpus striatum. After it had been in formalin some time Dr. Hadfield dissected the specimen, and said that it was an old hydatid cyst which had apparently dried and shrivelled up.

I have given the history of the case at some length because it is quite open to anyone to believe that this was one of ordinary idiopathic epilepsy, with the choroid hydatid as a coincidence. From the nature of the early attacks, the temperament of the patient between them, the absence of any symptoms of a fit or of *petit mal* from the age of two till she was forty, and the fact that though she had such a series of attacks shortly after her admission she had had none for many months before her death and had been so much more agreeable and amenable, I am led to believe that the hydatid cyst was the cause of the seizures through its pressure on the corpus striatum. If that be so, it supports a belief that not only may seizures indistinguishable from true epileptic attacks be caused by irritation of the striate body, but that the irritation which causes the fits may also give rise to that peculiarly irritable temperament which we so often meet in epileptics.

Whatever view may be taken of this particular case, there appears, at any rate, a fair amount of indirect evidence in favour of the view that the impulses which start the epileptic seizure originate in a subcortical centre, most probably the corpus striatum. This would account for the very rapid spread of the convulsion to all the muscles of the body, and for the similarity of the seizures of general paralysis and of pressure on the corpus striatum to those of the ordinary epileptic fit; whereas, if it is held that the epileptic convulsion is cortical in origin, it is difficult to account for the difference between the Jacksonian seizure which is known to be cortical and the attack in idiopathic epilepsy.

One other fact appears to have considerable bearing on this subject. All who have had much to do with epileptics must have noticed in many cases that the character of the voice is peculiarly modified, being uninflected and wooden in type. Scripture has described this condition of voice in the epileptic, and maintains that by taking a record of the speech vibrations he is able to diagnose any case of epilepsy, as the character is quite impossible of imitation by the non-epileptic. Kinnier Wilson, in discussing progressive lenticular degeneration and comparing its symptoms with those of paralysis agitans, mentions that in early cases of the former disease, as also in the latter, the character of the voice is altered. He says: "The patient's utterance is monotonous and sometimes dysarthric. To use a picturesque French expression: 'Il a perdu le chanson du lange.'"

It can hardly be a coincidence that an alteration in the tone of voice should occur in these two diseases, which are known to be due to affection of the corpus striatum, and also in epilepsy.

In what way is the corpus striatum concerned in connection with the motor system? The optic thalamus, besides being the relay ganglion in the course of the afferent sensory nerves, is also a centre for the association of the special senses, notably of sight and touch, whereby our elementary perceptions of objects are formed. Our perception of the size, position and form of those objects is dependent upon our sixth or muscular sense. Just as the senses of sight and touch have to be associated with each other, so has the muscle sense to be associated with them. As Herbert Spencer says: "There cannot be co-ordination of many stimuli without some ganglion through which they are brought into relation." And in the case of our sixth or muscle sense, with its enormous number of incoming stimuli associating it with all the other senses, that ganglion is the neo-striate part of the corpus striatum.

We have seen that it is intimately connected with the motor system, and that in lesions of parts of it there is frequently loss of muscle sense association evidenced by various forms of aphasia. We think in terms of muscle sense; in other words, every idea is an incipient muscular contraction. Hughlings Jackson in his Lumleian Lectures said: "When a healthy person thinks of doing something (has an idea of movement), I consider that what occurs physically is slight discharge not only of sensory but also of motor arrangements of his highest centres, there is nascent movement, slight discharge of the very same motor nervous arrangements of the highest centres which, if more strongly discharged, would, by intermediation of middle and lowest centres, produce the actual movement."

In paralysis agitans a common and distressing symptom is the inability to commence certain movements, it may be to get up out of a chair or start walking.

In aphasics it is common for the patient to forget words in the middle of a sentence, or to be unable to name objects at sight. Hughlings Jackson in his Croonian Lectures mentions the fact that aphasics can sometimes use and protrude the tongue which is not paralysed, but cannot put it out if asked to do so, though they evidently understand what is said to them, and will sometimes put the hand to the mouth to try and pull the tongue forward. In these cases there is no paralysis of motion, for the muscles can be used freely, and the required action is sometimes performed by the patient when not asked to do so. The inability is not due to an aboulia, for that is an affection of will power. It is not negativism, for the patients are perfectly willing and wish to perform the act required. In all these cases it would appear to be due to the inability to form the idea.

When a person wishes to rise from his seat or to perform any act those sensori-motor cells which will be concerned must first be mildly stimulated, giving rise to the idea. Upon further stimulation the idea becomes the act. In the absence of fibres and cells carrying impulses to those groups of cells in the sensori-motor region the idea cannot be aroused, or in other words a mental picture cannot be produced. These association impulses arise from various parts and in various ways. In the case of paralysis agitans they are aroused by the sight of another person whom he wishes to greet, or by the sound of someone requesting him to rise, and the same applies to the patient who cannot name articles at sight or protrude his tongue at will. In all these cases the failure is due to the inability of the association-currents to reach and excite those groups of sensori-motor cells which will be concerned in the movement, and if the corpus striatum be the centre for the association of muscle sense with all the others, lesions in its several parts will account for the various forms of aphasia, as word blindness, word deafness, etc.

It has been held for some time by many authorities that the corpus striatum is not connected with the cerebral cortex by direct fibres. Kinnier Wilson and Victor Horsley in 1914 carried out a number of carefully-planned and interesting experiments on the corpus striatum in apes, every precaution being taken to prevent injury to surrounding structures, and the results at which they arrived may be summed up as follows: (1) No direct fibres could be traced to the cortex or to the spinal cord; (2) there are no apparent fibres between the corpora striata of the two sides; (3) fibres from the caudate nucleus and putamen pass direct to the globus pallidus; (4) fibres from the globus pallidus pass in the thalamic fasciculus through the lower part of the internal capsule to end in the thalamus; (5) another strand, the strio-subthalamic or ansa lenticularis, leaves the globus pallidus and divides into two parts. One passes to the corpus subthalamicum, the other to the red nucleus. From this it seems that though impulses from the striate body do not pass by a direct route to the cortex they can, and no doubt do, by the more indirect path, namely, from caudate nucleus and putamen to the globus pallidus, thence viâ the thalamic fasciculus to the thalamus, whence they are relayed on to the cortex; or after leaving the globus pallidus they may pass in the ansa lenticularis and so reach the cortex viâ the red nucleus or the corpus subthalamicum and thalamus.

Wilson is evidently convinced that impulses from the corpus striatum reach the cortex even if by an indirect route, for he says in his paper on progressive lenticular degeneration, when discussing the probable cause of tremor: "It is conceivable, however, that the absence of the normal steadying influence of the lenticular nucleus may make itself felt viâ the strio-thalamic fibres on the external parts of the optic thalamus and so on the intermedio-precentral and precentral cortex." Weston Hurst in 1926 showed that the vessel walls in the globus pallidus in fifty out of a hundred cases of chronic nervous disease were infiltrated with iron salts. Barton White published the results of his investigations in thirty cases of mental disorder, showing a percentage of fifty-four with similar changes in the vessel walls. Hadfield examined the brains of thirty-two cases dying either from accidents or ordinary diseases not associated with mental or chronic nervous disorder, with a similar result, and says "that pallidal siderosis is probably the expression of a slow evolutionary atrophy, affecting the nuclei in at least 60 per cent. of people over the age of thirty."

The palæo-striate body is developed from the rhinencephalon; it contains large, probably motor cells, and its fibres myelinate before those of the neo-striate body.

From all this it appears probable that the globus pallidus is the ganglion in which those reflex actions originate (through the association of sensory impulses, chiefly visual and somatic, with the motor cells) which are necessary for the preservation of the species either by escape from enemies or by obtaining of food, these movements being purely reflex. But in animals with a more highly-developed consciousness, in whom after the first few months of life ideas are formed by the association of the other senses with the muscle sense and consequent stimulation of the sensori-motor cells, ideas which have to be transformed into actions many times before the corresponding reflexes are established, this palæo-striate body is only of use before this higher consciousness has developed, *i.e.* in the early part of their existence, chiefly for such reflex movements as sucking, feeding and swallowing. Later, when the putamen and caudate nucleus have become the centres for muscle-sense associations the palæo-striate body gradually deteriorates, and is used chiefly as a passage for the impulses which originate in these later developed nuclei to the cortex and other parts of the central nervous system. The fact that the neo-striate body, consisting of the putamen and caudate nucleus, is developed later, both phylogenetically and ontogenetically, appears to support this view.

As to the various mental states of the epileptic before and immediately following the seizures, also his temperament in the intervals, are these the consequences or merely the concomitants of the fits? That they are due directly to the seizures appears the more probable, for in other forms of epilepsy, migraine, etc., states of mental alienation do not appear to be more frequent than in the non-epileptic part of the community, and, contrary to what was thought at one time, states of acute mental trouble are not as common following *petit mal* as they are in those who suffer from the major attack. In fact, it is rare for a patient to be admitted to an asylum suffering only from the former; although, in the history of those who have had to be placed under restraint, it often transpires that they have suffered from attacks of *petit mal* for some years before the major seizures commenced, and that, as a rule, these attacks have continued at intervals for a considerable period before the mental state has necessitated the removal of the patient to an institution. What, then, is the probable explanation?

Bevan Lewis, in his text-book of mental diseases, when speaking of states of mental depression, points out very clearly that our ideas of the space attributes of the body are dependent largely on our sixth or muscular sense. He explains that the muscle sense concerned in these concepts is derived chiefly from the movement of the eyes, that the sense of these muscular contractions lapses eventually in consciousness as a pure muscular sensation, and that in states of mental depression this sense which has lapsed rises again into consciousness, giving rise to a feeling of effort which

(being unusual) at once suggests to the mind the notion of resistance, and so engenders ideas of suspicion and persecution. According to this author the muscular sense is the fundamental element of thought.

If those sensori-motor cells, the stimulation of which, as Dr. Hughlings Jackson suggests, gives rise to our ideas, are being constantly irritated by stimuli reaching them from an unstable centre, they will be maintained in a state of constant hypertension, ready to act too quickly, and this condition may easily account for the extreme irritability and impulsive conduct of epileptics in the intervals between their seizures.

Owing to the increased stimuli arising a short time before the fit, they will be in a state of still greater tension, and the conduct will become altered in consequence, giving rise to those conditions of sub-acute mania, increased suspicion with delusions, and sullen, dangerous moroseness so commonly met with at that time, so that their condition between, before and during the fits will be accounted for by the excessive irritation of these sensori-motor cells by impulses arising from an unstable ganglion (which, I submit, is the neo-striate body).

Hughlings Jackson has explained very clearly that the conditions of acute mania, stupor, automatism and semi-coma immediately following the seizures are to be explained as due to the temporary paralysis in varying degree of these same cells, due to exhaustion by the excessive discharge.

Can all this have any bearing on the treatment of the disease ? The various parts of the nervous system are very selective in their choice of drugs. Those which have been our stock remedies in epilepsy have been the bromides, which apparently act directly on the cortical cells, depressing their activity and so rendering them less liable to discharge. If drugs can be found (I believe luminal to be one) which act directly on the basal ganglia, reducing their irritability, the unwonted and excessive impulses which originate there in epileptics will be reduced or stopped, so relieving the cortex from their excessive irritation. By these means the seizures may be reduced in number and strength, the mental condition improved, as, indeed, we find so frequently the case, and it will be unnecessary to depress our patients with the bromides, which, to be of any use, must be given in large doses, with the result that the appetite is affected, skin eruptions produced, and that highly-developed, though most unstable organ, the cortex, caused to deteriorate and "a desolation created which we call peace."

To sum up. From the evidence before us, clinical, anatomical and psychological, it would appear that :—

1. Ordinary *grand mal* is produced by impulses which arise in the neo-striate body and act upon the cortex.

2. The mental condition of epileptics immediately preceding and in the intervals between the seizures is due to irritation of cortical sensori-motor cells by impulses originating in the striate body.

3. The neo-striate body, consisting of the putamen and caudate nucleus, is the centre for the association of the muscle sense with the other senses.

And as a corollary that—

4. Drugs which act primarily on the basal ganglia in epilepsy are to be preferred to those which act directly on the cortex, as being more efficient and less likely to damage the more highly-developed but less definitely organized cortical cells.

